# Changes in glycemic variability, gastric emptying and vascular endothelial function after switching from twice-daily to once-weekly exenatide in patients with type 2 diabetes: a subpopulation analysis of the twin-exenatide study

**DOI:** 10.1186/s12902-022-00932-9

**Published:** 2022-01-11

**Authors:** Jun Inaishi, Yoshifumi Saisho, Yuusuke Watanabe, Tami Tsuchiya, Hironobu Sasaki, Tatsuhiro Masaoka, Hiroshi Itoh

**Affiliations:** 1 Division of Endocrinology, Metabolism and Nephrology, Department of Internal Medicine, Keio University School of Medicine, Tokyo, Japan; 2 Center for Preventive Medicine, Keio University School of Medicine, Tokyo, Japan; 3 Department of Internal Medicine, Keio University School of Medicine, 35 Shinanomachi, Shinjuku-ku, 160-8582 Tokyo, Japan; 4 Division of Gastroenterology and Hepatology, Department of Internal Medicine, Keio University School of Medicine, Tokyo, Japan

**Keywords:** Type 2 diabetes, GLP-1 receptor agonist, Continuous glucose monitoring, Gastric emptying

## Abstract

**Background:**

We investigated the changes in blood glucose fluctuation, gastric emptying, and vascular endothelial function by switching from an exenatide twice-daily formulation (BID) to a once-weekly formulation (QW) since the evaluation of postprandial glucose excursion and glycemic variability (GV) by continuous glucose monitoring (CGM) after switching was lacking.

**Methods:**

Twenty-nine patients with type 2 diabetes treated with exenatide BID were included in this study and switched to exenatide QW for 24 weeks. GV assessed by CGM, gastric emptying (by ^13^ C-acetate breath test) and vascular endothelial function (by reactive hyperemia - peripheral arterial tonometry) were evaluated at baseline and 24 weeks after switching.

**Results:**

HbA1c decreased significantly from the baseline to week 24, while postprandial glucose levels after breakfast and dinner significantly increased (both *P* <0.05). However, the increases in GV indices were modest and not statistically significant at week 24. Vascular endothelial function was also not significantly changed after switching (*P* >0.05). Gastric emptying was significantly accelerated at week 24 (T_max_ 83.4 ± 12.1 min vs. 58.2 ± 16.4 min) (*P* <0.001) and correlated with increased postprandial glucose levels after breakfast and dinner (both *P* <0.05).

**Conclusions:**

Despite the increase in postprandial glucose associated with accelerated gastric emptying after switching from exenatide BID to QW, change in GV was modest and no significant deterioration in vascular endothelial function was observed after switching. These results support the superiority of treatment with exenatide QW over exenatide BID in clinical practice; however, attention should be paid to the monitoring and management of postprandial glucose levels when selecting exenatide QW.

**Trial registration:**

Clinical trial registry number; UMIN000016390 and jRCTs031180320.

Approval date of Registry and the Registration: December 12, 2014.

**Supplementary Information:**

The online version contains supplementary material available at 10.1186/s12902-022-00932-9.

## Background

Many new anti-diabetic medications for diabetes have been developed and marketed, including incretin glucagon-like peptide-1 (GLP-1) receptor agonists (GLP-1RAs), in the past two decades. It has been shown that GLP-1RAs reduce cardiovascular events in addition to lowering body weight and HbA1c in a recent meta-analysis [1]. For the management of type 2 diabetes (T2DM), the American Diabetes Association recommends treatment with GLP-1RAs for patients with established atherosclerotic cardiovascular disease, indicators of high risk, or chronic kidney disease [2]. However, the heterogeneity of the benefits on cardiovascular events among GLP-1RAs remains unclear [1], and it is necessary to clarify the characteristics of each GLP-1RA.

According to the differences in pharmacokinetic/pharmacodynamic profiles, GLP-1RAs are categorized as short- or long-acting compounds [3]. Exenatide, one of the GLP-1RAs, was developed based on exendin-4 [4], and there are two formulations of exenatide, a twice daily (BID) formulation as a short-acting GLP-1RA, and a once weekly (QW) formulation as a long-acting GLP-1RA. We have previously reported that switching from exenatide BID to exenatide QW reduced HbA1c and fasting plasma glucose, and improved beta cell function and treatment satisfaction in Japanese patients with T2DM [5]. Our and other studies also reported increased postprandial hyperglycemia after switching, using self-monitoring of blood glucose (SMBG) [5–7]; however, they lacked evaluation of postprandial glucose excursion and glycemic variability (GV) by continuous glucose monitoring (CGM).

Here, in this subpopulation analysis of the Twin-exenatide study [5], we evaluated the change in GV measured by CGM after switching from exenatide BID to exenatide QW, and its relation to gastric emptying and vascular endothelial function in Japanese patients with T2DM.

## Methods

This study was carried out with the approval of the Keio University Institutional Review Board for Clinical Research (20140298, approval date: Dec 12, 2014), and registered as trial numbers UMIN000016390 and jRCTs031180320. Before enrollment, all participants provided written informed consent to the investigators.

### Study design and subjects

**Fig. 1:**
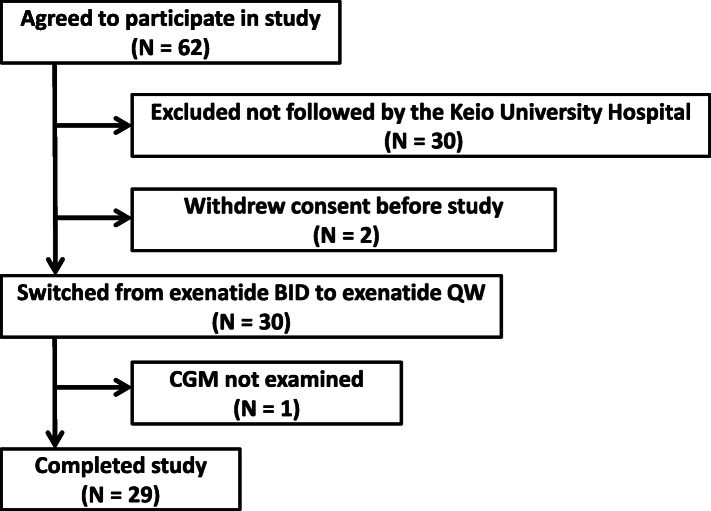
Disposition of study participants

The Twin-exenatide study was an investigator-initiated, prospective, single-arm, multicenter study. The details of this trial have been described in a previous report [5]. Adult 62 subjects who had been treated with exenatide BID (either 10 or 20 µg daily) for at least 3 months for T2DM were enrolled. The patients were switched from exenatide BID to exenatide QW (2 mg once-weekly) for 24 weeks. During the study at the Keio University Hospital, in addition to body weight, blood pressure and fasting blood and urine samples, CGM, ^13^ C-acetate breath test and reactive hyperemia - peripheral arterial tonometry (RH-PAT) were assessed at baseline (week 0) and week 24 and subjects who completed these assessments were included in this subpopulation analysis. Figure 1 shows the disposition of the participants in this study. Thirty patients not followed by the Keio University Hospital were excluded since CGM, 13 C-acetate breath test and RH-PAT were conducted only in the Keio University Hospital. Two patients withdrew consent before the trial and one patient did not undergo CGM. The characteristics of the remaining 29 patients who completed this study are shown in Table 1.

**Table 1:** Characteristics of study participants

N (male/female)	29 (28/1)
Age (years)	55 ± 12
Height (m)	1.68 ± 0.07
Weight (kg)	95.1 ± 32.7
BMI (kg/m^2^)	33.3 ± 10.6
Duration of diabetes (years)	12 ± 6
FPG (mg/dl)	164 ± 52
HbA1c (%)	7.4 ± 1.4
eGFR (ml/min/1.73m^2^)	75.1 ± 23.8
Hypertension (%)	86.2
Dyslipidemia (%)	72.4
CVD (%)	27.6
Concomitant anti-diabetic medication	
None (%)	13.8
SU (%)	55.2
Metformin (%)	79.3
TZD (%)	24.1
AGI (%)	10.3
SGLT2 inhibitor (%)	6.9

*FPG* fasting plasma glucose, *eGFR* estimated glomerular filtration rate, *CVD* cardiovascular disease, *SU* sulfonylurea, *TZD* thiazolidinedione, *AGI* α-glucosidase inhibitor, *SGLT2* sodium-glucose cotransporter 2

### Continuous glucose monitoring

CGM was performed using an iPro2 (Medtronic Japan Co., Ltd., Tokyo, Japan) for 5 days under blinded glucose levels, and patients were asked to conduct SMBG four times a day for calibration and record the time of meals during CGM measurement. We evaluated CGM indices for GV as follows; standard deviation (SD) of glucose, coefficient of variation (CV) of glucose, and mean amplitude of glycemic excursion (MAGE). The definitions and interpretation of each index were described in previous reports [8, 9] and recent review [10]. The SD glucose is influenced by the mean glucose level, while the CV glucose is calculated from the SD and mean blood glucose. The MAGE is the average of absolute differences between glucose peaks and nadirs. Time-in-range (TIR; percentage of time within the target range of 70–180 mg/dL), time-above-range (TAR; >180 mg/dL) and time-below-range (TBR; <70 mg/dL) were also evaluated. TIR is not a GV index, but it is widely recognized as a clinically appropriate target and has been reported that GV and TIR correlated with each other [10, 11].

### ^13^C-acetate breath test

This test was conducted as reported previously [12, 13]. Participants were asked to fast during more than 8 h before the test, and ingested a liquid diet (Racol, Otsuka, Japan) providing 200 kcal/200 ml, mixed with the test diet consisting of ^13^ C-acetate (100 mg). They exhaled air into a breath sampling bag as follows: prior to diet (0) and after 5, 10, 15, 20, 30, 40, 50, 60, 75, and 90 min. The ^13^CO_2_ value (‰) in breath was measured using an infrared spectrophotometer (POCone, Otsuka, Japan). The peak time (T_max_) of ^13^CO_2_ excretion was evaluated, and T_max_ >90 min was defined as 90 min in this study.

### Reactive hyperemia - peripheral arterial tonometry

Vascular endothelial function was evaluated by RH-PAT using an EndoPAT2000 (Itamar Medical, Israel). RH-PAT were performed in spine position after an overnight fast. This system measures digital volume changes accompanying pulse waves in a finger probe. The principle of RH-PAT and the calculation method of reactive hyperemia index are described elsewhere [14, 15]. Briefly, a blood pressure cuff was placed around the arm of subjects, and the opposite arm was defined as a control. After the pulse amplitude at baseline was measured, the cuff was inflated to 60 mmHg above systolic blood pressure or 200 mmHg for 5 min. After cuff deflation, the pulse amplitude was measured for 5 min.

### Laboratory measurements

Blood and urine samples were obtained after an overnight fast and before taking any medications. HbA1c and glycated albumin (GA) were measured by HPLC and enzymatic methods, respectively. Adiponectin was measured by latex agglutination turbidimetry. High-sensitivity C-reactive protein was measured by nephelometry. Urinary 8-hydroxy-2´-deoxyguanosine (8-OHdG) and 8-isoprostaglandin F2a (8-isoPGF2α) were measured by enzyme-linked immunosorbent assays, as previously reported [5].

### Statistical analysis

All statistical analyses were performed using the Statistical Package for the Social Sciences version 24 (IBM, USA). The level of significance was set at 5% (*P*<0.05). Data are presented as mean ± SD for continuous variables, and as proportion for categorical data in the tables. Paired t-test or Wilcoxon test was used to analyze changes in parameters from week 0 to week 24, and unpaired t-test or Mann-Whitney U test was used to compare differences in parameters between two groups. Spearman’s correlation coefficient was used to assess the association between two variables.

## Results

### Change in glycemic profile measured by CGM

**Fig. 2:**
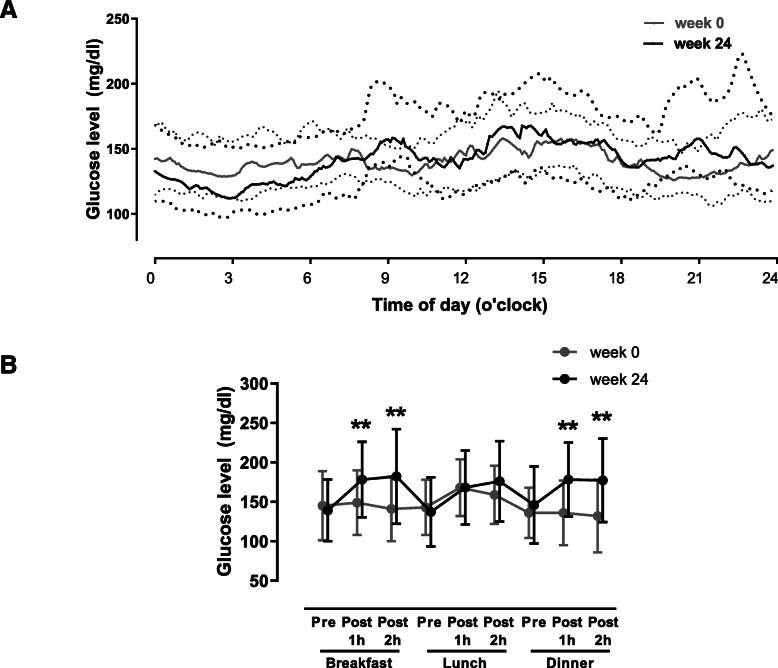
Median glucose levels measured by CGM throughout 24 h at week 0 and week 24 (**A**). Gray and black lines show levels at week 0 and week 24, respectively. The thick line in the middle shows the median, and the lines below and above the median show 25th and 75th percentiles, respectively. Changes in 1 and 2 h postprandial glucose excursions assessed by CGM (**B**). **P < 0.01 versus baseline (week 0)

The daily glycemic profile assessed by CGM and changes in postprandial glucose levels are shown in Fig. 2 A and B. In week 24 compared to week 0, glucose levels at 1 and 2 h post-breakfast (149 ± 41 vs. 186 ± 55 mg/dl and 141 ± 41 vs. 194 ± 68 mg/dl) and 1 and 2 h post-dinner (136 ± 41 vs. 184 ± 52 mg/dl and 132 ± 46 vs. 185 ± 61 mg/dl) were significantly increased (all *P* <0.01). Changes in CGM indices after switching from exenatide BID to exenatide QW are shown in Table 2. Despite the increase in postprandial glucose after switching, the increases were modest and there was no significant difference in GV indices, TIR and mean glucose assessed by CGM at week 24 (all *P* >0.05).

**Table 2:** Changes in CGM indices, ^13^ C-acetate breath test (T_max_) and vascular endothelial function (reactive hyperemia index) during study

Parameter	Baseline	Week 24	*P* value
HbA1c (%)	7.4 ± 1.4	7.1 ± 1.3	0.04
Fasting plasma glucose (mg/dl)	163.8 ± 52.1	143.2 ± 46.9	0.03
GA (%)	17.2 ± 3.9	16.8 ± 4.1	0.19
Weight (kg)	95.1 ± 32.7	95.3 ± 32.4	0.71
CGM indices			
Mean glucose (mg/dl)	152.0 ± 50.8	153.7 ± 47.8	0.83
SD glucose (mg/dl)	33.6 ± 15.2	36.0 ± 14.3	0.28
CV glucose (%)	21.6 ± 4.9	23.6 ± 7.1	0.11
MAGE (mg/dl)	67.0 ± 27.1	71.1 ± 29.4	0.30
Time in range (%)	78.3 ± 26.9	72.1 ± 29.2	0.17
Time above range (%)	21.0 ± 27.3	25.9 ± 30.1	0.27
Time below range (%)	0.7 ± 1.7	2.0 ± 3.9	0.12
T_max_ (min)	83.4 ± 12.2	58.2 ± 16.4	<0.001
Reactive hyperemia index	1.76 ± 0.39	1.84 ± 0.47	0.21
Adiponectin (µg/ml)	7.1 ± 3.4	7.3 ± 3.9	0.52
High-sensitivity CRP (mg/dl)	0.155 ± 0.170	0.209 ± 0.197	0.06
Urinary 8-OHdG (ng/mg creatinine)	7.9 ± 3.1	8.0 ± 3.2	0.75
Urinary 8-isoPGF2α (pg/mg creatinine)	439.2 ± 225.8	395.0 ± 113.8	0.32

*CGM* continuous glucose monitoring, *GA* glycated albumin, *SD* standard deviation, *CV* coefficient of variation, *MAGE* mean amplitude of glycemic excursions,　*8-OHdG* 8-hydroxy-2’-deoxyguanosine, *8-isoPGF2α* 8-isoprostaglandin F2α

### Changes in gastric emptying, vascular endothelial function and inflammation/oxidative stress markers

Table 2 and Supplementary Table 1 show the change in each parameter after switching from exenatide BID to exenatide QW. HbA1c and fasting plasma glucose were reduced (7.4 ± 1.4% vs. 7.1 ± 1.3% and 164 ± 52 mg/dl vs. 143 ± 47 mg/dl, both *P* <0.05) and gastric emptying (T_max_) evaluated by ^13^ C-acetate breath test was accelerated (83.4 ± 12.2 min vs. 58.2 ± 16.4 min, *P* <0.001) at week 24. There was no significant change in reactive hyperemia index by RH-PAT at week 24 (1.76 ± 0.39 vs. 1.84 ± 0.47, *P* = 0.21). Inflammation/oxidative stress markers (adiponectin, high-sensitivity CRP, urinary 8-OHdG and urinary 8-isoPGF2α) were not significantly changed at week 24 (all *P* >0.05) in this subpopulation analysis.

### Associations among CGM indices, gastric emptying, vascular endothelial function and inflammation/oxidative stress markers

To elucidate the associations among CGM indices, gastric emptying, inflammation/oxidative stress markers and clinical parameters, we conducted a pooled analysis of week 0 and 24 (Table 3 and supplementary Table 2). As a result, gastric emptying was significantly correlated with MAGE, TIR, TAR and postprandial glucose levels at breakfast and dinner (all *P* <0.05, Table 3). Moreover, there were significant correlations between change in gastric emptying and changes in MAGE, TIR, TAR, GA and body weight from week 0 to week 24 (all *P* <0.05, Supplementary Tables 3 and 4).

**Table 3:** Associations between ^13^ C-acetate breath test (T_max_), vascular endothelial function (reactive hyperemia index) and inflammation/oxidative stress markers and CGM indices in pooled data of week 0 and week 24

	Δ2h post-breakfast	Δ2h post-dinner	SD glucose	CV glucose	MAGE	Time in range	Time above range	Time below range
T_max_ (min)	-0.470**	-0.450**	-0.255	-0.095	-0.275*	0.351*	-0.310*	0.038
Reactive hyperemia index	-0.028	0.243	0.123	0.024	0.056	-0.212	0.164	-0.102
Adiponectin (µg/ml)	0.250	0.048	0.067	0.238	0.148	0.060	-0.086	0.131
High-sensitivity CRP (mg/dl)	0.007	0.110	0.222	0.056	0.126	-0.312*	0.223	0.088
Urinary 8-OHdG (ng/mg creatinine)	0.165	0.012	0.264*	0.274*	0.296*	-0.223	0.175	0.197
Urinary 8-isoPGF2α (pg/mg creatinine)	0.233	0.159	0.247	0.180	0.213	-0.181	0.190	-0.029

*CGM* continuous glucose monitoring, *SD* standard deviation, *CV* coefficient of variation, *MAGE* mean amplitude of glycemic excursions, *8-OHdG* 8-hydroxy-2’-deoxyguanosine, *8-isoPGF2α* 8-isoprostaglandin F2α, Δ2h post-breakfast; glucose level at 2 h after breakfast – pre-breakfast, Δ2h post-dinner; glucose level at 2 h after dinner – pre-dinner. *P < 0.05 and **P < 0.01

There were significant correlations between high-sensitivity CRP and TIR, and between urinary 8-OHdG and SD glucose, CV glucose and MAGE (all *P* <0.05, Table 3), suggesting the possible effects of GV or TIR on inflammation and oxidative stress. On the other hand, there were no significant correlations between reactive hyperemia index or adiponectin and CGM indices.

## Discussion

The present study characterized the changes in blood glucose fluctuation, gastric emptying, and vascular endothelial function after switching from exenatide BID, a short-acting GLP-1RA, to exenatide QW, a long-acting GLP-1RA, in patients with T2DM. The main findings of this study were that (1) postprandial glucose levels measured by CGM increased after switching, which were correlated with accelerated gastric emptying, and (2) the increase in GV assessed by CGM was, however, modest and not statistically significant and there were no significant changes in vascular endothelial function as well as inflammation/oxidative stress markers after switching.

It has been reported that long-acting GLP-1RAs primarily reduce fasting plasma glucose levels through enhancing insulin secretion [3], while short-acting GLP-1RAs primarily reduce postprandial glucose levels by delaying gastric emptying [16]. In this study, postprandial glucose levels after breakfast and dinner measured by CGM were increased after switching from exenatide BID to exenatide QW, in line with the results of previous studies using SMBG [5–7].

On the other hand, despite the increase in postprandial glucose after switching, we found that the increase in GV assessed by CGM was modest and not statistically significant. TIR and TBR were also not significantly changed after switching. This could be due to the improvement of overall glucose levels and reduction in glucose fluctuation especially during night time, accompanied by improvement of beta cell function after switching. Unexpectedly, mean glucose levels assessed by CGM were not significantly changed at week 24 despite the reducing HbA1c at week 24. As shown in the original Twin-exenatide study [5], this subpopulation analysis also showed that fasting plasma glucose was reduced significantly at week 24. Since HbA1c is reported to more closely correlate with fasting plasma glucose than with postprandial glycemic excursion [17], improving fasting plasma glucose, rather than GV, TIR or mean glucose levels, by switching to exenatide QW might have affected the reduction of HbA1c. The short period of CGM monitoring, i.e., five days rather than 14 days, and the effects of dose reduction in concomitant medications such as sulfonylurea during the study might also cause the discordance between HbA1c and mean glucose level assessed by CGM in this study. There were also no significant changes in vascular endothelial function as well as inflammation/oxidative stress markers after switching.

In this study, gastric emptying was significantly accelerated after switching, in line with previous studies [16, 18]. Gastric emptying was significantly associated with postprandial glucose excursion after breakfast and dinner, indicating that the increase in postprandial glucose levels after switching was due to the recovery of gastric emptying. Furthermore, change in gastric emptying was correlated with changes of GA and body weight as well as MAGE. Thus, our findings indicate the significance of gastric emptying in postprandial glucose excursion, GV and weight control during GLP-1RA therapy.

GV has been shown to increase reactive oxygen species production and cardiovascular complications [19–22]. Although inflammatory/oxidative markers were not significantly changed after switching from exenatide BID to exenatide QW, oxidative stress markers such as high-sensitivity CRP and urinary 8-OHdG were associated with some GV indices and TIR in the pooled analysis of this study. We have also reported a significant increase in urinary 8-isoPGF2α level after switching in the original Twin-exenatide study [5]. In the Exenatide Study of Cardiovascular Event Lowering (EXSCEL) study [23], treatment with exenatide QW failed to reduce cardiovascular events compared with placebo. A recent study also showed no difference in carotid plaque progression between treatment with exenatide QW and placebo [24]. Taking these results together, the non-significant cardiovascular protective effects with exenatide QW could be due to insufficient suppression of oxidative stress, although other long-acting GLP-1RAs have shown significant cardiovascular protection [25].

The efficacy and safety of exenatide QW have been shown in a number of trials [26–31]. In addition, treatment with exenatide QW has been reported to result in reduction in fasting plasma glucose, HbA1c, incidence of hypoglycemia, and gastrointestinal adverse events, and improvement of beta cell function and treatment satisfaction compared to exenatide BID [5–7, 32, 33], and these results have been confirmed in patients of the Twin-exenatide study [25]. This subpopulation analysis of the Twin-exenatide study also showed no significant deterioration in GV and endothelial function after switching from exenatide BID to exenatide QW, supporting the superiority of use of exenatide QW over exenatide BID in clinical settings. However, attention should be paid to monitoring postprandial glucose levels when selecting exenatide QW, and prompt management of postprandial glucose excursion is needed.

The strength of this study is that this was the first study precisely evaluating change in GV after switching from exenatide BID to exenatide QW by using CGM. However, there are several limitations of this study. First, this study did not directly compare the two drugs because of its single-armed design. However, our results can be applied to clinical practice in the real-world since this study was carried out in general practice. Second, the statistical power might be limited by the small sample size of this subpopulation analysis. Third, the participants of this study tended to be obese and predominantly male. Thus, the results may not be applicable to those without obesity, females, or other ethnicities.

## Conclusions

This subpopulation analysis of the Twin-exenatide study demonstrated modest, but not significant, deterioration in GV after switching from exenatide BID to exenatide QW, although postprandial glucose was increased after switching, associated with accelerated gastric emptying. Moreover, there was no significant change in vascular endothelial function after switching. Together with the results of the Twin-exenatide study [5], these findings support the superiority of treatment with exenatide QW over exenatide BID in clinical practice. However, attention should be paid to the monitoring and management of postprandial glucose levels when selecting exenatide QW.

## Supplementary information

**Additional file 1.**

## Data Availability

The datasets used and/or analyzed during the current study, are available from the corresponding author on reasonable request.

## Abbreviations

T2DM: type 2 diabetes
QW: once-weekly
BID: twice daily
GLP-1: glucagon-like peptide-1
HbA1c: glycated hemoglobin
FPG: fasting plasma glucose
GA: glycated albumin
eGFR: estimated glomerular filtration rate
8-OHdG: 8-hydroxy-2’-deoxyguanosine
8-isoPGF2α: 8-isoprostaglandin F2α
CGM: continuous glucose monitoring
RH-PAT: reactive hyperemia - peripheral arterial tonometry
