# Supplementary material for: Changes in glycemic variability, gastric emptying and vascular endothelial function after switching from twice-daily to once-weekly exenatide in patients with type 2 diabetes: a subpopulation analysis of the twin-exenatide study
Source: BMC Endocr Disord. 2022 Jan 11;22:20. doi: 10.1186/s12902-022-00932-9 (PMC8751111; doi:10.1186/s12902-022-00932-9)
Supplement: Supplementary file 1 — Additional file 1. [file 12902_2022_932_MOESM1_ESM.docx]

Supplementary Table 1. Changes in other clinical parameters during study.

| Parameter | Baseline | Week 24 | P value |
| --- | --- | --- | --- |
| BMI (kg/m^2^) | 33.4 ± 10.6 | 33.4 ± 10.4 | 0.81 |
| 1,5-AG (μg/ml) | 7.7 ± 6.3 | 8.1 ± 6.1 | 0.40 |
| Insulin (mU/l) | 15.5 ± 14.3 | 15.7 ± 12.4 | 0.94 |
| HOMA-IR | 4.4 (2.4-6.9) | 3.9 (2.7-7.5) | 0.25 |
| HOMA-β (%) | 50.1 (22.3-76.3) | 54.2 (32.8-124.7) | 0.007 |
| C-peptide (ng/ml) | 3.52 ± 2.22 | 3.58 ± 2.06 | 0.82 |
| C-peptide index | 2.31 ± 1.67 | 2.76 ± 2.03 | 0.06 |
| Creatinine (mg/dl) | 0.90 ± 0.27 | 0.94 ± 0.28 | 0.26 |

1,5-AG; 1,5-anhydro-D-glucitol.Supplementary Table 2. Associations between ^13^C-acetate breath test (T_max_) and clinical parameters in pooled data of week 0 and week 24.

|  | T_max_ (min) | P value |
| --- | --- | --- |
| HbA1c (%) | -0.180 | 0.18 |
| Fasting plasma glucose (mg/dl) | -0.051 | 0.71 |
| GA (%) | -0.122 | 0.37 |
| Weight (kg) | -0.207 | 0.13 |
| BMI (kg/m^2^) | -0.172 | 0.21 |

Supplementary Table 3. Associations between change in ^13^C-acetate breath test (T_max_) and changes in clinical parameters from week 0 to week 24.

|  | ΔT_max_ (min) | P value |
| --- | --- | --- |
| ΔHbA1c (%) | -0.358 | 0.06 |
| ΔFasting plasma glucose (mg/dl) | -0.248 | 0.20 |
| ΔGA (%) | -0.579 | <0.01 |
| ΔWeight (kg) | -0.382 | 0.04 |
| ΔBMI (kg/m^2^) | -0.399 | 0.04 |

Supplementary Table 4. Associations between changes in ^13^C-acetate breath test (T_max_), vascular endothelial function (reactive hyperemia index) and oxidative stress markers and changes in CGM indices from week 0 to week 24.

|  | Δ Glucose level at 2 hours post-breakfast – pre-breakfast | Δ Glucose level at 2 hours post-dinner – pre-dinner | ΔSD glucose | ΔCV glucose | ΔMAGE | ΔTime in range | ΔTime above range | ΔTime below range |
| --- | --- | --- | --- | --- | --- | --- | --- | --- |
| ΔT_max_ (min) | -0.246 | -0.139 | -0.260 | 0.332 | -0.375* | 0.409* | 0.460* | 0.358 |
| ΔReactive hyperemia index | -0.142 | 0.063 | 0.101 | 0.077 | 0.077 | -0.150 | 0.141 | -0.037 |
| ΔAdiponectin (µg/ml) | -0.162 | -0.222 | 0.026 | 0.205 | -0.104 | 0.078 | -0.127 | 0.264 |
| ΔHigh-sensitivity CRP (mg/dl) | -0.027 | 0.344 | 0.169 | -0.001 | 0.264 | -0.202 | 0.147 | -0.047 |
| ΔUrinary 8-OHdG (ng/mg creatinine) | 0.165 | 0.012 | -0.064 | 0.196 | -0.073 | 0.223 | 0.175 | 0.197 |
| ΔUrinary 8-isoPGF2α (pg/mg creatinine) | -0.063 | -0.011 | -0.009 | 0.239 | -0.043 | 0.267 | 0.280 | 0.130 |

CGM; continuous glucose monitoring, SD; standard deviation, CV; coefficient of variation, MAGE; mean amplitude of glycemic excursions, 8-OHdG; 8-hydroxy-2’-deoxyguanosine, 8-isoPGF2α; 8-isoprostaglandin F2α. *P < 0.05 and **P < 0.01.
